# Intentional replantation and dental autotransplantation of mandibular posterior teeth: Two case reports

**DOI:** 10.1097/MD.0000000000035822

**Published:** 2023-11-17

**Authors:** Yao Wang, Maria Hofmann, Sabine Ruf, Jian Zhang, Qiuju Huang

**Affiliations:** a Department of Orthodontics, Faculty of Medicine, Justus Liebig University Giessen, Giessen, Germany; b Department of Paediatric Dentistry, Medical Center for Dentistry, University Medical Center Giessen and Marburg, Giessen, Germany; c Department of Oral and Maxillofacial Surgery, Wuxi Stomatology Hospital, Wuxi, China; d Department of Endodontics, Wuxi Stomatology Hospital, Wuxi, China.

**Keywords:** apical surgery, case report, dental autotransplantation, intentional replantation, root canal treatment

## Abstract

**Background:**

Intentional replantation and dental autotransplantation are 2 similar techniques both involving atraumatic tooth extraction, visualization of the root, and replantation. They are considered as the last resort for unsalvageable teeth. The author aims to describe 2 mandibular posterior teeth with serious periapical lesions which are resolved by intentional replantation and dental autotransplantation, respectively.

**Case summary:**

In case 1, a 45-year-old male patient received root canal treatment because of a cracked mandible right first molar with periapical lesions. An endodontic file was separated in the apical third of the mesiolingual root canal. After conventional canal filling of the other root canals, the molar was atraumatically extracted. The separated instrument was removed, the mesiolingual root received a retrograde filling and the molar was replanted. At the 3-month follow up, the patient was asymptomatic and the X-ray picture showed no detectable root resorption and ankylosis. In case 2, a 29-year-old woman reported discomfort during occlusal loading after a root canal treatment and a coronal restoration of the mandibular right first molar. Radiographs showed a low-density shadow in the mesial apical and in the root furcation area of the mandibular first molar so the patient was diagnosed as chronic periapical periodontitis. After the removal of the affected tooth, the extraction socket was thoroughly debrided and irrigated. The intact mandibular right third molar with similar dimensions was extracted by minimally invasive procedure and transplanted. The donor tooth was fixed by a fiber-splint for 1 month and a root canal treatment was performed 2 weeks after surgery. After 1 year, clinical and radiographical examination revealed functional and periodontal healing.

**Conclusions:**

These 2 reports present the successful management of intentional replantation and dental autotransplantation. Both procedures are recommended after nonsurgical endodontic treatment, especially when apical microsurgery is not an option, for example because of difficult accessibility or patient preference.

## 1. Introduction

Apical surgery is a reliable treatment for patients with persistent or recurrent periapical lesions.^[1]^ However, apical surgery is not appropriate in some cases for numerous reasons, including difficult accessibility, anatomic structure, aesthetic consideration and patient acceptability. Intentional replantation (IR) offers an alternative for those cases with limitations. It is defined as an endodontic manipulation or repair after atraumatic tooth extraction, followed by the replantation of the tooth into the original socket.^[2]^ Some unrestorable teeth can be replaced by a healthy autogenous tooth from another region, usually a third molar or a supernumerary tooth.^[3,4]^ This procedure is known as dental autotransplantation (DAT). IR and DAT share a common biological basis, the regeneration of the periodontal ligament (PDL) and the pulp revascularization. Nevertheless, the PDL and the pulpal cells could be impaired during the procedure, which leads to root resorption and ankylosis.^[5]^ The destruction of regenerative cells could be the reason for controversial prognosis.^[6,7]^ This article deals with the management of 2 complicated endodontic cases using IR and DAT, respectively. The aim is to discuss the factors which could affect the clinical outcome of IR or DAT and to offer treatment guidance for clinicians.

## 2. Case reports

This case report has been written according to CARE guidelines published in 2013 and 2017 in the Journal of Clinical Epidemiology. Our provisional plan of using IR and DAT as clinical treatments was approved by the Ethics Committee of Wuxi Stomatology Hospital.

### 2.1. Case 1: intentional replantation

**Figure 1.**
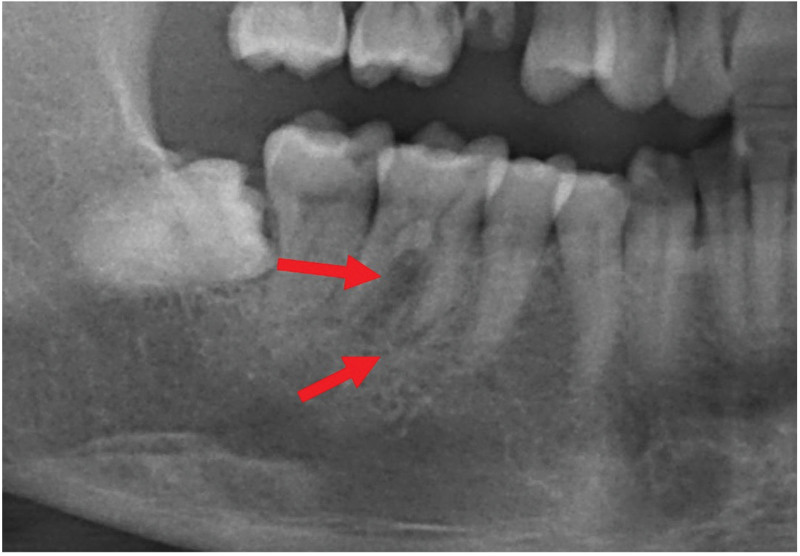
Preoperative panoramic radiograph illustrating a radiolucent lesion around the mesial root and root furcation of tooth 46 (see red arrows).

A 45-year-old male patient in good general health was referred to the Department of Endodontics at Wuxi Stomatology Hospital in Wuxi, China. The patient complained of intermittent dull pain in the lower molar teeth during the last month. A history of biting on a hard object was reported by the patient. Clinical examination indicated a crack of the mandible right first molar (tooth 46) which also showed a negative response to electric pulp testing. Percussion sensitivity was observed but the tooth did not present increased mobility. The panoramic radiographic examination revealed an apical low-density shadow invading the root furcation area (Fig. 1). A typical periapical lesion was diagnosed. The initial treatment plan was a basic periodontal treatment, followed by a root canal treatment (RCT) and a coronal restoration.

**Figure 2.**
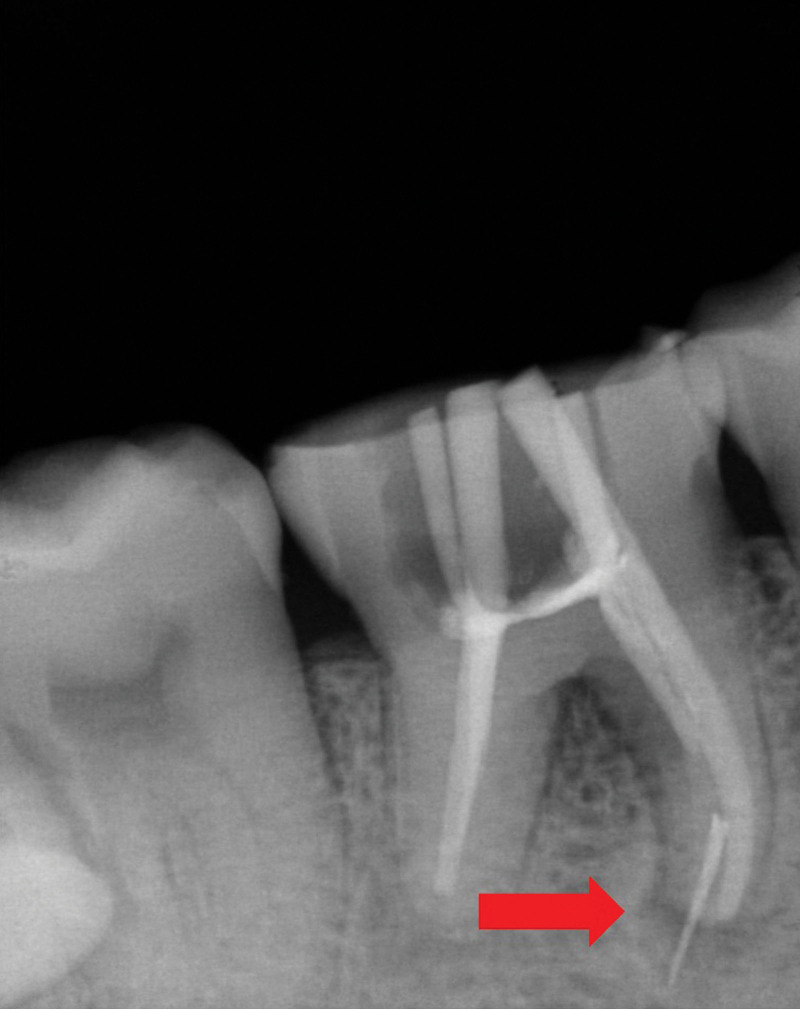
Periapical radiograph showing the separated instrument extending beyond the root apex.

**Figure 3.**
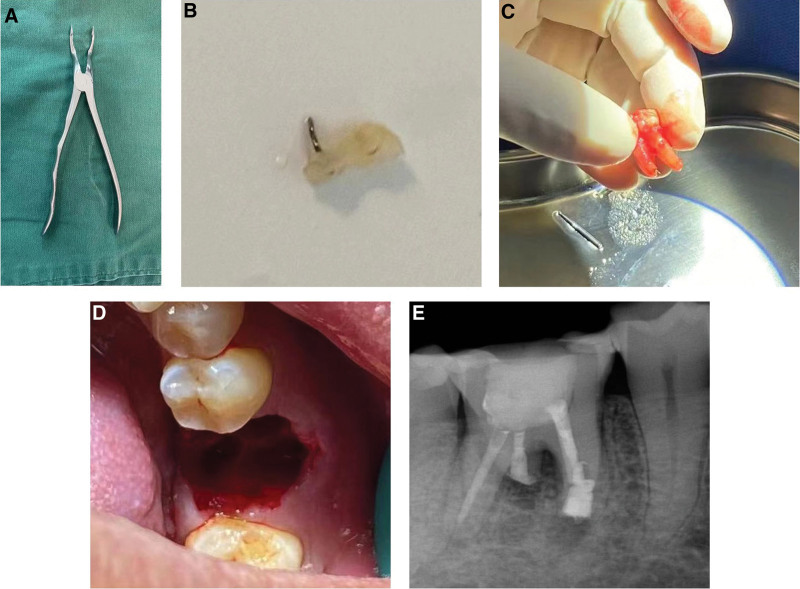
Surgical procedure: (A) atraumatic dental forceps were used to extract the tooth; (B) apical fragment with separated file is removed; (C) extracted tooth is kept wet with saline; (D) extraction socket is fresh and intact after removal of granulation; (E) immediate post-operative periapical radiograph revealed that the tooth was in place.

**Figure 4.**
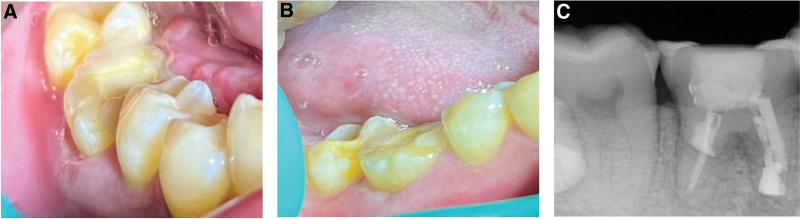
Clinical and radiographic examination with favorable prognosis at 3-month follow-up.

During the root canal preparation, a nickel-titanium (NiTi) rotary instrument (M3 Pro-gold rotary files, United Dental Group, China) separated. Diagnostic radiography illuminated the fractured part of the file extending 4 mm beyond the apex of the mesiolingual root (Fig. 2). The attempts to remove or by-pass the broken instrument were unsuccessful. Thus, the clinician adapted the treatment plan and decided to pursue an intentional replantation. An informed consent was obtained after informing the patient of the risks and the benefits of the procedure. Firstly, the other 3 root canals were filled with single-cone technique using gutta-percha (Dentsply Sirona) and iRoot SP sealer (Innovative BioCeramix, Canada). Then, tooth 46 was atraumatically extracted under local anesthesia using forceps without any intraoperative complication. A curettage to remove granulation tissues in the socket, followed by rinsing with sterile saline was performed. Afterwards, tooth 46 was kept moist with saline and an apical resection was carefully performed in vitro to remove the fractured tip of the instrument. The apical third of the remaining mesiolingual root was filled retrogradely by using iRoot BP plus (Innovative BioCeramix, Canada). Lastly, the tooth was replanted into the extraction socket and the coronal two thirds of the root canal was filled with gutta-percha (Dentsply Sirona, USA). A light-cured resin (3M, USA) was utilized to fill the pulp chamber (Fig. 3). Tooth 46 was fixed to the adjacent teeth by means of a flowable resin (3M, USA), followed by an occlusion adjustment. The patient was advised not to chew hard food with his right posterior teeth for 3 months. After 3 months, neither pain upon palpation/percussion nor increased tooth mobility was observed. The radiographic examination revealed no root resorption or ankylosis (Fig. 4). The patient was satisfied with therapeutic outcome.

### 2.2. Case 2: tooth autotransplantation

**Figure 5.**
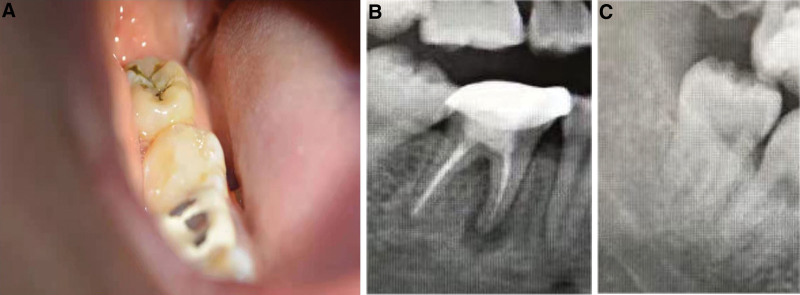
Imaging data before treatment: (A) clinical examination showed a poor PFM crown restoration on tooth 46; (B) preoperative panoramic radiographic indicating a severe peri-apical lesion invading the mesial root and the root furcation of tooth 46; (c) tooth 48 is intact and morphologically similar to tooth 46. PFM = porcelain fused to metal.

**Figure 6.**
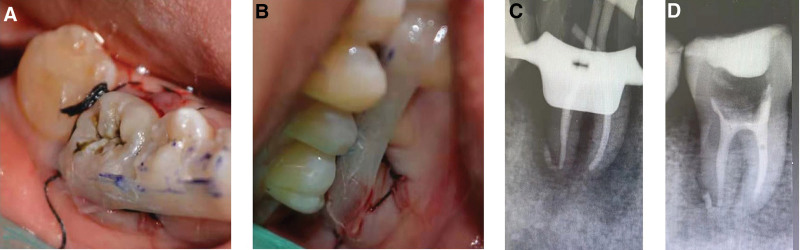
(A) Fiber-splint fixation of donor tooth with 45 and 44; (B) occlusion adjusted to eliminate premature contacts and cuspal interferences; (C and D) root canal treatment performed 1 month after surgery.

**Figure 7.**
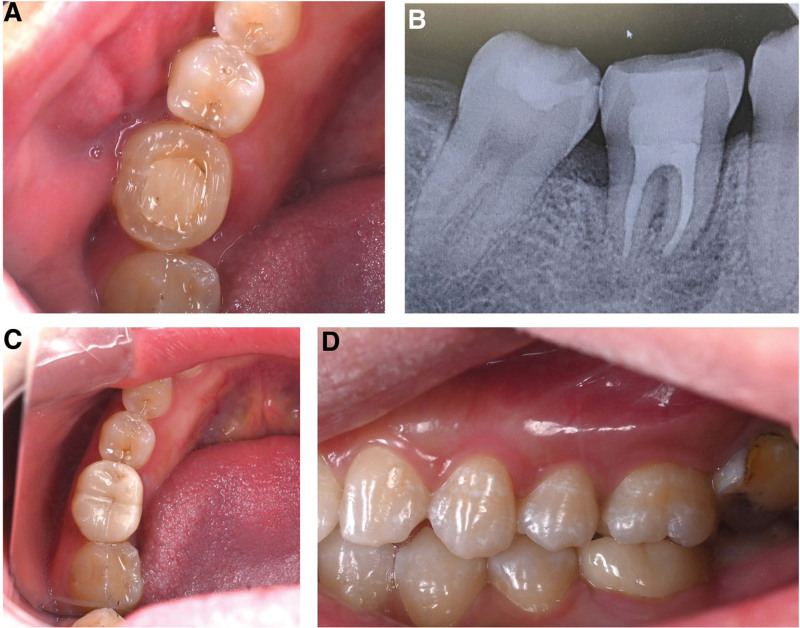
One-year postoperative examination: (A) intraoral examination showing soft tissue healing; (B) radiographic image revealing low-density shadow vanish; (C and D) occlusal and lateral view of final prosthesis.

A systemically healthy 29-year-old female patient with discomfort of the lower right first molar was referred to the Department of Endodontics at Wuxi Stomatology in Wuxi, China. A root canal treatment had been performed 3 to 4 years ago in a private general dental practice. Intraoral examination revealed a porcelain fused to metal (PFM) crown restoration with a marginal gap on tooth 46. The tooth was percussion sensitive. The preoperative panoramic radiograph revealed a severe periapical lesion involving the root furcation but no appreciable bone resorption. The patient was diagnosed as chronic periapical periodontitis but refused root canal retreatment. An implant restoration was refused for financial reasons. As the patient’s lower right third molar (tooth 48) had a similar crown and root morphology as tooth 46 (Fig. 5), a DAT was conducted after receiving informed consent. Tooth 46 was extracted, and the extraction socket was thoroughly curetted and rinsed to remove granulation tissue. Moreover, tooth 48 was atraumatically extracted and immediately placed in the recipient socket. The donor tooth was fixed to tooth 45 and tooth 44 by a fiber-splint. An occlusal adjustment was performed to prevent occlusal trauma. Two weeks later, a root canal treatment of the donor tooth was conducted (Fig. 6). After 1 year, tooth 46 doesn’t exhibit percussion sensitivity or mobility. Radiographic image indicated peri-radicular lesion reduction, and there was no sign of inflammatory or replacement resorption. After then, patient received tooth preparation and ceramic fixed prosthesis was delivered subsequently (Fig. 7). The patient was satisfied with final clinical outcome.

## 3. Discussion

Fracturing of endodontic instruments is a common complication during root canal treatment. Reported separation rates range between 0.25% and 6% for stainless steel instruments and between 1.3% and 10.0% for NiTi rotary instruments.^[8]^ Even though it is acceptable to retain separated instrument parts within a root canal for some patients with only pulpitis, Strindberg^[9]^ reported that a fractured instrument could lead to a poorer prognosis in the presence of periapical lesions. File parts are less likely to be removed by nonsurgical procedure if the fragment is positioned beyond the root canal curvature,^[10]^ like in case 1. However, the procedure of apical surgery poses risks due to the anatomical proximity to the inferior alveolar nerve and the dense and thick mandible buccal cortical bone.^[11]^ Considering these limitations, IR presents a less invasive alternative to conventional apical surgery. It allows the clinician to perform an extraoral retrograde filling which is simpler, quicker, and more economical.^[12]^ In addition, multiple materials have been deployed as root-end filling materials, such as zinc oxide-eugenol cement, intermediate restorative material, glass ionomer cements, amalgam, and mineral trioxide aggregate cement. The filling material used in this case, iRoot BP Plus, is a hydrophilic calcium silicate-based bioceramic material with excellent mechanical properties, sealing ability, and antibacterial activity, which promotes osteo-/odontogenic differentiation of mesenchymal stem cells.^[13,14]^

DAT is a similar technique which is only considered when affected teeth are unrestorable. Compared to IR, it is usually more challenging because of incompatible anatomy between the recipient site and the donor tooth, which has to be considered before surgery. A socket that is too small could increase the compressive force between the donor tooth and the socket wall, which impairs the PDL cells on the surface of the donor root, while an oversized socket will reduce the initial stability of the donor tooth. Thus, to acquire an appropriate recipient alveolus that is 10% larger than the donor tooth, the clinician would have to adjust it during surgery.^[15]^ On the other hand, in contrast to IR, a tooth replanted in a DAT is usually an intact tooth with a fresh and healthy pulp. Thus, RCT is not always necessary, especially for young donor teeth with incomplete root formation.^[15]^ The apex diameter is considered an essential factor that impacts the success of DAT because it is associated with pulp revascularization. It is recommended to select a donor tooth with apical foramina of more than 1 mm, which is beneficial for the invasion of blood capillaries.^[16]^ In case 2, given the age of the patient and the narrow apex diameter, we performed a preventative RCT after 4 weeks. Lastly, an adequate post-operative splint is crucial for periodontal healing. The splinting period could be extended if the donor tooth does not match the socket.^[17]^ However, excessively rigid or constant splinting could increase the risk of ankylosis. Thus, a flexible fiber-splint and a splinting period of less than 6 weeks are recommended in most cases.^[2,18]^

Some scholars attempted to improve IR and DAT procedures. Asgary^[19]^ employed a modified combination of triple antibiotics to arrest external root resorption after DAT. A defective recipient socket is a challenge for PDL healing. Yao^[20]^ placed Bio-oss bone powder (0.25 g; Geistlich, China) into the apical bone defect to enhance bone regeneration. Lescure^[21]^ acquired a satisfying clinical outcome using leukocyte- and platelet-rich fibrin (L-PRF) to improve PDL healing. To minimize PDL cell damage, the extra-alveolar time of the donor tooth should be strictly limited to a maximum of 15 minutes.^[2,22]^ Thus, a necessary RCT should be performed before or after surgery. If possible, saline or cell culture medium should be used to maintain the activity of PDL cells.^[23]^ In addition, it was reported that a 3D printed donor replica and guide plate were used to shorten surgery time and to assist donor emplacement.^[4,24]^

The limitation of this report is the short follow-up period. Even though no detectable complication was reported in the cases, further clinical trials are needed to determine long-term outcome and complication risks. Before its long-term prognosis is confirmed, IR and DAT can only serve as treatments of last resort for unsalvageable teeth.

## 4. Conclusions

IR and DAT are viable treatment alternatives of apical surgery when conventional endodontic treatment is unapplicable.

## Author contributions

**Conceptualization:** Yao Wang, Qiuju Huang.

**Data curation:** Yao Wang, Jian Zhang, Qiuju Huang.

**Formal analysis:** Yao Wang.

**Methodology:** Yao Wang, Jian Zhang.

**Project administration**: Qiuju Huang.

**Supervision:** Qiuju Huang.

**Validation:** Yao Wang, Maria Hofmann, Sabine Ruf, Qiuju Huang.

**Visualization:** Yao Wang.

**Writing—original draft:** Yao Wang.

**Writing—review & editing:** Yao Wang, Maria Hofmann, Sabine Ruf, Qiuju Huang.
